# Janus Kinase Mediates Faster Recovery From Sevoflurane Anesthesia Than Isoflurane Anesthesia in the Migratory Locusts

**DOI:** 10.3389/fphys.2022.806746

**Published:** 2022-03-30

**Authors:** Zongyuan Ma, Jialin C. Zheng, Tianzuo Li, Zhongcong Xie, Le Kang

**Affiliations:** ^1^Beijing Institutes of Life Science, Chinese Academy of Sciences, Beijing, China; ^2^Center for Translational Neurodegeneration and Regenerative Therapy, Shanghai Tenth People’s Hospital, Tongji University School of Medicine, Shanghai, China; ^3^Department of Anesthesiology, Beijing Shijitan Hospital, Capital Medical University, Beijing, China; ^4^Department of Anesthesia, Critical Care and Pain Medicine, Massachusetts General Hospital and Harvard Medical School, Boston, MA, United States

**Keywords:** Migratory Locusts, Janus Kinase, Anesthesia, Recovery, RNA Interference

## Abstract

Inhalation anesthetics isoflurane and sevoflurane have been widely used in clinical practice for anesthesia. However, the molecular mechanisms underlying the faster recovery from sevoflurane anesthesia than isoflurane anesthesia remain largely undetermined. Herein, we use RNA-seq, RNA interference, quantitative real-time PCR and western blotting to explore the mechanisms of recovery from isoflurane and sevoflurane anesthesia in the migratory locusts. Although the migratory locusts show similar anesthetic responses to these two chemicals in corresponding half-maximal effective concentrations (EC50s), the recovery from sevoflurane anesthesia is significantly faster than that for isoflurane anesthesia after 30 min of anesthetic exposure. Transcriptome analysis shows that those transcripts involved in cytoskeletal components, Janus kinase (JAK) pathway and cuticle protein are differentially expressed in locust brains in response to isoflurane and sevoflurane. RNAi knockdown confirms that *Actin*, *Myosin-like protein 84B* (*Mlp84B*), *JAK* and cuticle protein *NCP56* do not affect anesthetic response of the locusts to these two chemical anesthetics. Moreover, *actin*, *Mlp84B* and *NCP56* do not affect differential recovery from isoflurane and sevoflurane anesthesia, whereas RNAi knockdown of *JAK* and its partner *STAT5B* does not affect anesthetic recovery from isoflurane but elongates recovery duration from sevoflurane anesthesia. Thus, JAK may mediate faster recovery from sevoflurane anesthesia than from isoflurane anesthesia in the migratory locust. This finding provides novel insights into the molecular mechanism underlying faster recovery from sevoflurane anesthesia than isoflurane anesthesia.

## Introduction

General anesthesia is a reversible condition induced by anesthetic drugs, and this condition shows loss of behavioral states of consciousness, amnesia, analgesia, and immobility underlying physiological stability. Although inhaled volatile anesthetics have been widely used in clinical practice for quick induction of general anesthesia (Brown et al., 2011), the recovery duration from anesthesia shows great variances among inhaled anesthetics (Misal et al., 2016; Vutskits and Xie, 2016), and underlying mechanisms of anesthetic recovery are not fully understood.

Isoflurane and sevoflurane are two of the most commonly used inhaled anesthetics in clinical operation. Isoflurane is a fluorinated ether with general anesthetic and muscle relaxant activities, whereas sevoflurane is a new fluorinated methylisopropyl ether inhalational anesthetic (Franks, 2008). Although isoflurane has an excellent safety record in clinical practice, sevoflurane shows the faster onset of anesthesia effects and ensures rapid awakening (Cantillo et al., 1997; Ebert et al., 1998). In clinical practice, sevoflurane anesthesia has a clinical advantage of maintaining stable hemodynamics and rapid recovery (Chen et al., 1998). In obese and geriatric patients, sevoflurane shows a faster recovery and greater respiratory safety as compared with isoflurane (Golembiewski, 2004; Gupta et al., 2004; Arar et al., 2005). In neurosurgery and prolonged open urological surgery, sevoflurane provides faster emergence than that of isoflurane (Peduto et al., 1998; Gauthier et al., 2002). Recovery of cognitive and psychomotor function seems to be faster and more complete after sevoflurane anesthesia than isoflurane anesthesia (Olthoff and Rohrbach, 1998; Schwender et al., 1998). On the other hand, in veterinary clinics, recovery duration is significantly shorter for sevoflurane anesthesia than that for isoflurane anesthesia in dog and avian operations (Kramer et al., 2008; Anjana et al., 2021). Thus, these previous studies have reported that postoperative recovery duration for sevoflurane anesthesia is shorter than that for isoflurane anesthesia.

The mechanisms by which faster recovery of sevoflurane anesthesia than isoflurane anesthesia have attracted great interests in anesthesia studies. Sevoflurane shows lower blood: gas partition coefficient than that of isoflurane, and this lower coefficient in sevoflurane may lead to the faster elimination kinetics than that of isoflurane. On the other hand, sevoflurane and isoflurane affect neurochemical transmission in brains during anesthesia recovery (Taylor et al., 2013; Westphalen et al., 2013; Aksenov et al., 2019). Sevoflurane affects metabolisms of dopamine and serotonin in recovery from anesthesia (Ogasawara et al., 1992, 1993). Activation of adenosine 5‘-monophosphate (AMP)-activated protein kinase (AMPK) pathway by sevoflurane accelerates the emergence from sevoflurane anesthesia (Finley, 2019). D-amphetamine may interact with D1/D5 dopamine receptors to induce the emergence from sevoflurane (Kenny et al., 2015; Pal et al., 2018). Activation of basal forebrain (BF) GABA*^SOM^* neurons facilitates isoflurane anesthesia, and induces longer recovery time after isoflurane anesthesia (Cai et al., 2021). Although neurotransmission of neurochemicals potentially mediates recovery from sevoflurane anesthesia and isoflurane anesthesia (Taylor et al., 2013; Westphalen et al., 2013; Aksenov et al., 2019), the molecular mechanism underlying the shorter recovery duration for sevoflurane than that for isoflurane anesthesia is not well understood until now.

The anesthesia and post-anesthesia studies in invertebrate model are critical for clarifying anesthesia and recovery mechanisms in the early phase of clinical studies. Fruit flies and worms are indeed paramount choices for invertebrate anesthesia study (Zalucki and van Swinderen, 2016; Fischer et al., 2018; Olufs et al., 2018). However, the migratory locust (*Locusta migratoria*) has its own specific traits that prompt us to perform exploratory and pioneering anesthesia study in this animal model. The migratory locust is an experimental model that has been widely used in invertebrate behavioral and neurobiological studies. This insect is easy for economically culturing in the laboratory. It has spiracle in each somite in abdomen, and its intricate tracheole respiratory system is compared favorably with tracheal respiratory system in mammals. The locust respiratory system makes it feasible for studies related with volatile general anesthesia. Moreover, its larger body size makes it much easier for behavioral phenotype observation during anesthesia. Its larger brain accommodates the injection of chemical compound for pharmacological anesthesia studies. More importantly, the larger brain makes the neurobiological electrophysiology manipulation more practical when studying neurophysiological mechanism underlying anesthesia and post-anesthesia recovery. On the other hand, although long evolutionary distance occurs between locusts and mammals or humans, evolution has produce physiological, cellular and molecular mechanisms that are conserved between locusts and humans (Kellogg and Shaffer, 1993), and locusts have been used for studying human diseases and sickness behavior (Siddiqui and Khan, 2020). Locust spreading depression study provides conserved clues for exploring production of human neurodegeneration diseases (Ayata and Lauritzen, 2015; Spong et al., 2016), and the conserved brain efflux activity makes promise for human pharmacological research (Al-Qadi et al., 2015). Furthermore, the conserved growth cone is translated for testing developmental neurotoxicity in humans (Bergmann et al., 2019). The conservative traits of neurophysiology and molecular mechanism between locusts and mammalian nervous system propel us to choose this insect as an animal model to study neurogenetic mechanism underlying anesthesia and post-anesthesia recovery.

In this study, we compared isoflurane- and sevoflurane-induced anesthetic effects on the migratory locusts and differential duration of recovery from two anesthetics. After the administrations of isoflurane and sevoflurane in the migratory locusts, RNA-seq analysis was performed to detect the resultant gene expression profiles in brains, and JAK mediates the faster recovery from sevoflurane anesthesia than isoflurane.

## Materials and Methods

### Animals

All experiments were performed using the fifth-stadium migratory locusts (*Locusta migratoria*). The locust colonies were maintained at the Institute of Zoology, Chinese Academy of Sciences, Beijing, China. These locusts were cultured in large boxes (40 cm × 40 cm × 40 cm) at a density of 200–300 locusts per container. This colony was maintained under a 14-h:10-h light: dark photocycle regime. The culturing temperature is maintained at 30°C ± 2°C by air-conditioner. These locusts are fed on fresh wheat seedling and dry wheat bran, and female and male locusts were both used for experimentation (Ma et al., 2011).

### Anesthesia

The fifth-stadium locusts after molting 3 or 4 days were randomly assigned to the control and anesthesia groups, and equal number of male and female locusts were assigned to each group. The locust with leg or antenna loss was not used for anesthesia study. On the day of anesthesia exposure, ten locusts from the anesthesia groups were put into a clear plastic box (15 cm × 15 cm × 10 cm) for one replicate and were left in the box for 5 min before anesthetic administration. Anesthesia was respectively induced by administration of sevoflurane and isoflurane (RWD Life Science, Shenzhen, China) with a calibrated vaporizer. Fresh air (21% O_2/_79% N_2_) was fed through the vaporizer at a constant flow rate of 2 L/min. We put a thermometer in the plastic box to monitor temperature during the locust anesthesia, and the temperature in this box for anesthesia exposure was about 30°C ± 2°C. The anesthetic concentrations (isoflurane: 0.2%, 0.4%, 0.6%, 0.8%, 1.0%, 1.2%, 1.4%, 1.6%, 1.8% and 2.0% and sevoflurane: 1.2%, 1.6%, 2.0%, 2.4%, 2.8%, 3.2%, 3.6%, 4.0%, 4.4%, 4.8%, 5.2% and 5.6%) in this chamber were continuously measured by gas monitor (Draeger, Germany). The gas monitor was calibrated at the zero scale by the technician from Draeger company before initiating this study. During the induction of anesthesia, vaporizer settings were adjusted to maintain the selected concentration of isoflurane and sevoflurane in the inspired gas mixture. We considered locusts to be full-anesthetized if they appeared immobile, and their legs, abdomen and antennae did not show obvious motion after mechanical touching by a brush pen (Supplementary Video 1). Thus, anesthesia in the locust is defined by the loss of body movement, and the absence of any responses in legs, abdomen and antennae after mechanical touching by a brush pen. The mechanical touching may avoid the confusion of anesthesia with hypnosis in evaluating anesthetized locusts. The control groups were also put in the same box with the same temperature, lighting conditions and carrier air. The experimental assistant helped to set the concentration of anesthetics, and the investigator independently noted the percentage of anesthetized locusts and duration of recovery from isoflurane and sevoflurane anesthesia. The investigators assessing anesthesia effects were blinded to the concentrations of anesthetics or fresh exposures. After a specific duration of exposure, anesthesia administration was discontinued and the migratory locusts were removed to stay in an empty culture box to recover from anesthesia (Supplementary Video 1). The time point for locust anesthesia recovery is when the locust stands on its legs after gross body movements (movements in legs, abdomen and antennae). When the locust stood on its legs after body movement, we immediately used a brush pen to assess whether this locust showed mechanical response to the brush stimulus and had recovered from anesthesia (Supplementary Video 1). The recovery duration is defined as the time interval from discontinuation of anesthetic delivery to standing of the locust on its legs after gross body movements. Ten timers were prepared to record recovery duration of each insect after discontinuing anesthetic exposure.

Moreover, anesthetics (isoflurane: 0.8%, 1.2%, and 1.6%; sevoflurane: 2.8%, 4.0% and 5.2%) were respectively delivered to the plastic box (15 cm × 15 cm × 10 cm) with the fifth-stadium locusts (10 locusts in per box for one replicate) for a 120-min treatment, and anesthesia rates were recorded after 10 min, 20 min, 30 min, 40 min, 50 min, 60 min and 120 min respectively to optimize the time duration for anesthesia. Concentrations of 0.2%, 0.4%, 0.6%, 0.8%, 1.0%, 1.2%, 1.4%, 1.6%, 1.8% and 2.0% were used for the EC50 curve for isoflurane, and concentrations of 1.2%,1.6%, 2.0%, 2.4%, 2.8%, 3.2%, 3.6%, 4.0%, 4.4%, 4.8%, 5.2% and 5.6% were used for the EC50 curve for sevoflurane. After anesthesia, the locusts were left in open clean air for natural recovery. To follow recovery of locusts from anesthesia, investigators removed the anesthetized locusts from the plastic box after exposure to the anesthetics. Once locusts stood on their legs after gross body movements and showed mechanical responses to brush open stimulus, these locusts were considered to recover from anesthesia.

### RNA Extraction and RNA Sequencing

Brains and thoracic ganglia (similar as spinal cord in vertebrates) are the target tissues of anesthetics. During anesthesia recovery in locusts, the recovery of brain activity may be related with recovery of perception of outer stimuli (olfaction, vision, etc.) and controlling of complex behaviors (including leg and abdomen movements), and the recovery activity in thoracic ganglia may be correlated with recovery of leg and abdomen movements. Thus, in this study, we collected brain tissue to better understand the mechanisms of anesthesia recovery. After the migratory locusts were treated with isoflurane (1.2%) or sevoflurane (3.2%) for 30 min, brain tissue of these insects were collected and stored at −80°C, ten brains were pooled together for one biological replicate. Three biological replicates were prepared for RNA extraction and sequencing. Total RNA was purified using TRIzol (Life Technologies, Carlsbad, CA, United States) according to the manufacturer’s protocol. DNA contamination was eliminated by DNase I (Qiagen, Germany). An Illumina HiSeq 2500 (Illumina, CA, United States) sequencing platform was used for RNA-seq. Adaptor contamination and low-quality reads were discarded from the raw data. Trinity software (version 2.0.10) was used for sequence assembly and alignment, and expression levels of transcripts were analyzed with Bowtie and RSEM modules in Trinity software (Grabherr et al., 2011). Differentially expressed transcripts (DETs) were identified using edgeR software (Robinson et al., 2010). Those transcripts with a fold change > 1.5 and *P* < 0.01 were selected as DETs. Finally, Blast2GO was used to annotate DETs and enrich pathways (Conesa et al., 2005). The raw reads of all samples are available from the NCBI SRA server (accession number: PRJNA717646).

### RNA Purification and Quantitative Real-Time PCR Assay

After extraction of total RNAs from brain tissues with TRIzol (Life Technologies, Carlsbad, CA, United States) according to manufacturer’s protocol, we performed reverse transcription using Moloney murine leukemia virus (M-MLV) reverse transcriptase (Promega, Madison, WI, United States). qRT-PCR was conducted using Roche Light Cycler 480 SYBR Green I Master (Roche, Indianapolis, IN, United States). The gene encoding ribosomal protein 49 (*RP49*) was used as the housekeeping gene for data normalization and estimation.

### RNA Interference

To inhibit the expression levels of JAK and STAT5B mRNA, we designed fragments for RNA interference (RNAi) and blasted these fragments against the *Locusta* genome database to detect sequence homologies and avoid non-specificity (Wang et al., 2014). To prepare and synthesize the double-stranded RNAs (dsRNAs), we chose a T7 RiboMAX Express RNAi system (Promega, Madison, WI, United States). Table 1 shows the primers for targeted fragment amplification and dsRNA synthesis. After injecting dsRNA (3 μl, 2.5 μg/μl) into the head cavity of the fifth-stadium locusts, we placed those injected locusts in their cages for an additional 3 days before confirming RNAi efficiency or anesthesia treatment. The RNAi efficiency was confirmed using quantitative real-time PCR (qRT-PCR) 3 days after injections (Guo et al., 2011; Ma et al., 2015).

**TABLE 1:** Primer sequences for qRT-PCR and RNAi knockdown.

Name	Forward primer (5′-3′)	Reverse primer (5′-3′)
*Actin* qPCR	*ACCACGGCTGAGCGAGAA*	GATACCGCACGATTCCAT
*Actin* RNAi	ACCACGGCTGAGCGAGAA	ACTTGCGGTGAACGATGC
*Mlp84B* qPCR	GACTCCACCAACTGCTCCG	AAGCAATCCCTGTGCCAAC
*Mlp84B* RNAi	CTGGCAAAGGGAACGATGT	CAGTTGTTGGTTTGGCATC
*NCP56* qPCR	TGCCCTTCCCCTACACCT	AGGGAGTCACCAGGGAGC
*NCP56* RNAi	TGCCCTTCCCCTACACCTAC	CGAAGGCAGTGTCAGGGAGT
*JAK* qPCR	GACATTGAGCCACAGTTAGTT	ATGGCTATCTCACGCTCAAA
*JAK* RNAi	TGACCATGCTGCTTCTTCAC	ATGGCTATCTCACGCTCAAA
*RP*–*49*	CGCTACAAGAAGCTTA AGAGGTCAT	CCTACGGCGCACTCTGTTG

### Western Blotting

The brain tissues were homogenized in the TRIzol reagent using a tissue grinding pestle. The protein homogenates of each tissue were mixed with 4 × NuPAGE™ LDS sample buffer containing 2% β-mercaptoethanol. The sample mixtures were boiled at 95°C for 5 min and then cooled down to the room temperature. After the protein mixture was centrifuged for 2 min at 12000 rpm, the supernatants were loaded on a 12% SDS PAGE gel. Proteins were electrophoretically separated at a voltage of 100 V for approximately 80 min until the dye reached the bottom. Typically, 80 μg of proteins from each tissue were loaded in each lane. Proteins were transferred to polyvinylidene difluoride membrane (Millipore, United States). After the membrane was blocked with 5% non-fat dry milk in phosphate-buffered saline containing 0.1% Tween-20 (PBST) for 1 h at room temperature, it was incubated with the primary antibody. The primary antibody was diluted with 5% milk in PBST as instructed by manufacturer and incubated at 4 °C overnight. We manufactured the polyclonal antibody against JAK in ABclonal company in China (ABclonal, Wuhan, China). We chose monoclonal mouse antibody GAPDH (Cat# CW0100M, CWBIO, Taizhou, China) as the positive control for normalization. The antibody against JAK were diluted 1:1000. Following an overnight incubation with three washes with PBST (5 min each), the membrane was incubated with a secondary antibody (1:3000) (Epienzyme, Shanghai, China) for 1 h at room temperature. We used the highly sensitive 5-bromo-4-chloro-3-indolyl phosphate/nitroblue tetrazolium substrate (Life Sciences, United States) to detect protein bands. Quantity One software (Bio-Rad Laboratories, Berkeley, United States) was used to calculate the intensity of WB signals, and the fold change in the protein level relative to the level of the corresponding controls was presented.

### Anesthesia Pharmacology

In previous studies, the JAK inhibitor AG490 and STAT5 inhibitor SC355979 respectively inhibit the function of JAK and STAT5 to protect the brains of migratory locusts with hypoxia exposure (Miljus et al., 2014). AG490 (Targetmol, Wellesley Hills, MA, United States) and SC355979 (Targetmol, Wellesley Hills, MA, United States) were dissolved in 3% DMSO with the concentrations of 200 mM and 80 mM, respectively. To further validate the functional roles of JAK and STAT5B in mediating differential anesthesia recovery from isoflurane and sevoflurane, we separately injected 1 μl of the JAK inhibitor AG-490 (200 mM) and 1 μl of the STAT5B inhibitor SC355979 (80 mM) in head cavities of the fifth-stadium migratory locusts. We chose the central position between two paired compound eyes to inject drug solutions into anterior of the locust brain using a micro-syringe, and injection depth was about 1-2 mm. To avoid the damage caused by micro-injection, we inserted tips of the micro-syringe in the direction from ventral to dorsal side of the locust head. We slowly injected the drug solution to make sure the solution diffused in head cavity as much as possible. The slow injection also kept the drug solution from running out of the pinhole (Guo et al., 2013). We then further analyzed their anesthetic responses and anesthesia recovery duration 1 h later.

### Statistical Analysis

Anesthetic differences between the controls and anesthetic-treated locusts were analyzed by Student’s *t*-test and one-way analysis of variance (ANOVA). The expression levels of the target transcripts were also analyzed by Student’s *t*-test. The recovery duration differences in RNAi knockdown were analyzed by Mann–Whitney *U* test, and the recovery durations of the migratory locusts from isoflurane (0.2% to 2.0%) and sevoflurane (0.8% to 5.6%) exposures were analyzed by the Kruskal-Wallis test. Normality testing (Shapiro-Wilk test) was performed prior to use of *t*-test and ANOVA. These statistical analyses were performed with SPSS 20.0 (IBM Corp., Armonk, NY, United States). G-test for independence is used for analyzing significant changes of the percentage of recovered and unrecovered locusts within 10 min after discontinuing isoflurane and sevoflurane anesthesia. EC50 curves were fitted with a non-linear regression model. Dose-response analysis was performed using SigmaPlot 12.5 (Systat Software, Inc., San Jose, CA, United States). Data were initially entered into Microsoft Office Excel 2017 (Microsoft Corporation, Redmond, WA, United States) and were subsequently imported into SigmaPlot. The dose values were imported as *x*-values, and the corresponding anesthesia data were entered as *y*-values. A variable slope sigmoidal E*^max^* model sigmoidal (four-parameter logistic) dose-response curve was fitted to the data for isoflurane and sevoflurane anesthesia, respectively.

## Results

### The Anesthetic Effects of Isoflurane and Sevoflurane in Migratory Locusts

**FIGURE 1:**
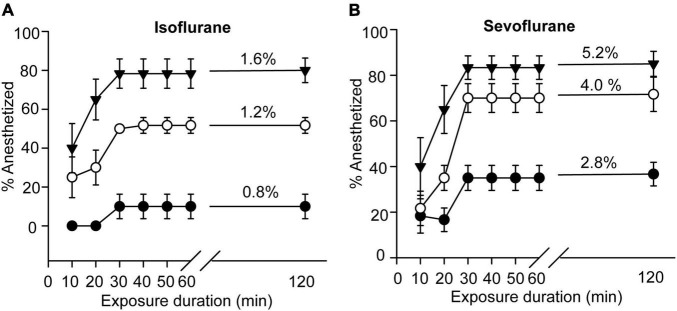
Anesthesia effects of isoflurane and sevoflurane on the migratory locusts. **(A)** Anesthetic response of the migratory locusts to isoflurane (0.8%, 1.2%, and 1.6%, *n* = 60 for each time point) after 120-min exposure interval. **(B)** Anesthetic response of the migratory locusts to sevoflurane (2.8%, 4.0%, and 5.2%, *n* = 60 for each time point) after 120-min exposure interval. One-way ANOVA analysis is used for analyzing anesthetic effect. The percentage of anesthetized locusts is shown as the mean ± SEM.

We first investigated the anesthetic effects of isoflurane and sevoflurane on the fifth-stadium migratory locusts. After exposure to isoflurane for 10 min, 20 min, 30 min, 40 min, 50 min, 60 min, and 120 min, the migratory locusts showed significant anesthesia response to concentrations of 0.8%, 1.2% and 1.6% (*F* = 5.00, *P* < 0.01 for 0.8%; *F* = 22.532, *P* < 0.001 for 1.2%; *F* = 18.63, *P* < 0.001 for 1.6%). The anesthesia rate did not change after 30 min of exposure to isoflurane (Figure 1A). On the other hand, at the concentrations of 2.8%, 4.0% and 5.2% for sevoflurane, the migratory locusts showed significant anesthesia responses after 10 min, 20 min, 30 min, 40 min, 50 min, 60 min and 120 min of sevoflurane exposure (*F* = 15.00, *P* < 0.01 for 0.8%; *F* = 17.87, *P* < 0.001 for 1.2%; *F* = 21.269, *P* < 0.001 for 1.6%) (Figure 1B). Moreover, the percentage of insects in sevoflurane anesthesia did not change after 30 min of exposure to all three concentrations of sevoflurane. Thus, the migratory locusts show striking responses to these two anesthetics, and 30 min of exposure to two anesthetic drugs marks a critical transition point for anesthesia.

**FIGURE 2:**
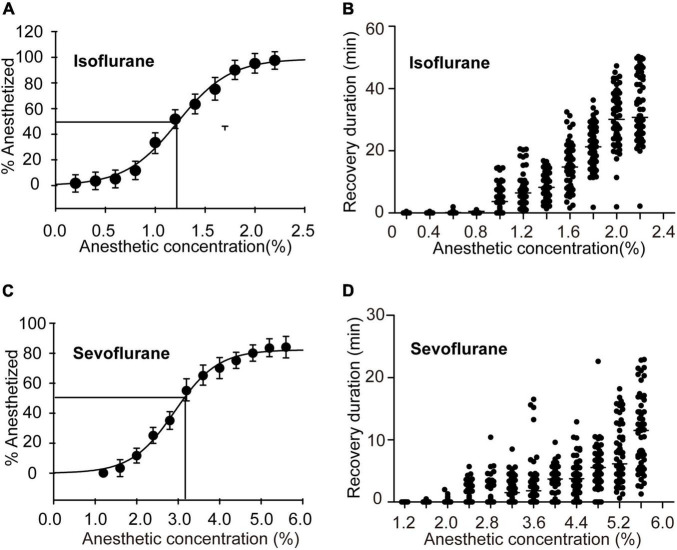
Half-maximal effective concentrations (EC50s) after 30 min of exposure and recovery durations after exposure to graded concentrations of anesthetics. **(A)** EC50 and the graded concentration-response curve for the migratory locusts exposed to isoflurane (*n* = 6 groups for each concentration, and 10 locusts in each group). **(B)** Recovery durations of the migratory locusts after exposure to the graded concentrations of isoflurane (*n* = 60 for each dosage, Kruskal-Wallis test). **(C)** EC50 and the graded concentration-response curve for the migratory locusts with sevoflurane exposure (*n* = 6 groups for each concentration, and 10 locusts in each group). **(D)** Recovery durations of the migratory locusts after the exposure to the graded concentrations of sevoflurane (*n* = 60 for each dosage, Kruskal-Wallis test). Percentage of anesthetized locusts is shown as the mean ± SEM. One-way ANOVA is used for analyzing anesthesia response, and recovery durations of the migratory locusts from graded concentrations of anesthetics were analyzed by Kruskal-Wallis test.

Considering that the anesthetized rate did not significantly change after 30 min of exposure to isoflurane and sevoflurane, we sought to investigate the graded concentration responses to the inhaled anesthetics for 30-min anesthesia and determine the EC50s of these two drugs in locusts, respectively. We firstly analyzed anesthetic responses of the migratory locusts to 30 min of isoflurane exposure at 11 graded concentrations from 0.20% to 2.20%. The results showed that anesthesia effect of the locusts was increased significantly with the increasing concentration of isoflurane (Chi-Square = 61.517, *P* < 0.001) (Figure 2A), suggesting a significant anesthetic response of this insect to isoflurane. We then performed non-linear regression analysis to predict the EC50, or the concentration that can induce immobility in 50% of the migratory locusts (non-linear regression, R^2^ = 0.9936, *P* < 0.001). These results showed that EC50 of isoflurane was approximately 1.2%. After investigating recovery duration of locusts in 30 min of isoflurane exposure, we found that anesthesia recovery duration tended to increase gradually with increasing concentrations of isoflurane (Chi-Square = 63.32, *P* < 0.001). Thus, when exposure to the higher concentrations of isoflurane, the migratory locusts may need more time to recover from anesthesia.

Furthermore, we investigated anesthetic responses of these locusts to the graded concentrations of sevoflurane (1.20% to 5.60%) and assessed the recovery duration after 30 min of sevoflurane exposure. The percentage of anesthetized locusts was increased significantly with the increasing concentration of sevoflurane (*F* = 54.726, *P* < 0.001) (Figure 2C). The EC50 of sevoflurane as analyzed by non-linear regression model was approximately 3.2% (non-linear regression: R^2^ = 0.9975, *P* < 0.001). The recovery duration was also increased significantly with increasing concentration of sevoflurane (Chi-Square = 65.272, *P* < 0.001) (Figure 2D). Therefore, the migratory locusts show concentration-dependent responses to anesthetics, and thereafter the recovery duration is increased corresponding to the increase of isoflurane and sevoflurane concentrations, respectively. Moreover, the locusts with isoflurane anesthesia (EC50, 1.2%) take about 20 min for complete recovery, whereas those insects with sevoflurane anesthesia (EC50, 3.2%) need about 10 min for complete recovery (Figure 2B), implicating the shorter duration of recovery from sevoflurane anesthesia than from isoflurane anesthesia.

### The Migratory Locusts Show Differential Recovery From Isoflurane Anesthesia and Sevoflurane Anesthesia

**FIGURE 3:**
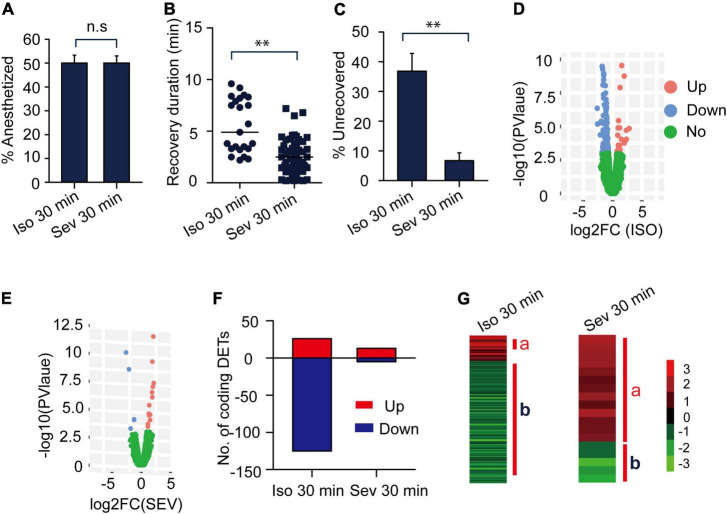
The gene expression profiles of transcripts responding to isoflurane (1.2%) and sevoflurane (3.2%) anesthesia. **(A)** The anesthesia rate of the migratory locusts responding to 30 min of isoflurane (1.2%) and sevoflurane (3.2%) (*n* = 6 groups, and 10 locusts in each group). Percentage of anesthetized locusts is shown as the mean ± SEM. **(B)** The recovery duration of the migratory locusts after 30 min of isoflurane (1.2%) and sevoflurane (3.2%) exposure (*n* = 38 for isoflurane; *n* = 60 for sevoflurane). **(C)** The percentage of unrecovered locusts with sevoflurane anesthesia is lower than those insects with isoflurane anesthesia within 10 min after discontinuing anesthesia. **(D)** The expression profiles of those transcripts in responding to isoflurane anesthesia. **(E)** The expression profiles of transcripts in responding to sevoflurane anesthesia. **(F)** Numbers of differentially expressed transcripts (DETs) in response to isoflurane and sevoflurane anesthesia. **(G)** The DETs induced by 30-min isoflurane and sevoflurane anesthesia were respectively clustered for analyzing gene expression patterns. Student’s *t*-test is used for analyzing anesthetic responses, and Mann–Whitney *U* test is used for analyzing the differences of recovery duration from anesthesia. G-test for independence is used for analyzing statistical significance of the differential proportions of unrecovered locusts with isoflurane and sevoflurane anesthesia within 10 min after discontinuing anesthesia. n.s *P* > 0.05; ^**^P < 0.01. JAK, Janus kinase; Iso, isoflurane; Sev, sevoflurane.

Although nearly 50 percent of this insect were anesthetized in 30 min of isoflurane exposure (1.2%) or 30 min of sevoflurane exposure (3.2%) (Figure 3A), they did not show obvious phenotype differences during exposure to isoflurane and sevoflurane. Considering that recovery is faster in sevoflurane anesthesia than in isoflurane anesthesia in fruit flies and in clinical practice (Peduto et al., 1998; Arar et al., 2005; Olufs et al., 2018), we intended to explore phenotypic differences during the recovery from isoflurane and sevoflurane anesthesia.

Almost all the locusts with sevoflurane anesthesia had recovered at 10 min after discontinuing anesthesia (Figure 2D), whereas the locusts with isoflurane anesthesia had not recovered completely during this time interval (Figure 2B). Thus, in this study, we chose this time (within 10 min after discontinuing anesthesia) to analyze phenotypic differences during the recovery from isoflurane and sevoflurane anesthesia. At the time interval of 10 min after discontinuing anesthesia, we recorded the recovery duration of every recovered locust for behavioral evaluation. We also counted the percentage of recovered and unrecovered locusts within 10 min to further compare their differential proportions in isoflurane and sevoflurane anesthesia. Thus, we chose the recovery duration and the proportion of unrecovered animals as two parameters to assess differential recovery of anesthesia within 10 min after discontinuing anesthesia.

In this study, the anesthesia recovery assay showed that the recovery duration of locusts with sevoflurane anesthesia was shorter than those with isoflurane anesthesia (Mann–Whitney *U* = 214, *P* < 0.01) (Figure 3B), about 37 percent of isoflurane-anesthetized locusts did not recover within 10 min after discontinuing anesthesia, and the proportion of the unrecovered locusts was significantly higher in isoflurane anesthesia than those in sevoflurane anesthesia (*G* = 14.06, *P* < 0.01) (Figure 3C). Thus, conforming to anesthesia recovery of sevoflurane and isoflurane in fruit flies and in clinical operations (Peduto et al., 1998; Arar et al., 2005; Olufs et al., 2018), the migratory locusts show shorter recovery duration in sevoflurane anesthesia than in isoflurane anesthesia.

### Isoflurane and Sevoflurane Anesthesia Induce Differential Expression Patterns of the Genes in Brains of the Migratory Locusts

Besides the different blood: gas coefficients of isoflurane and sevoflurane may contribute to differential recovery in isoflurane and sevoflurane anesthesia, the genetic and physiological effects of isoflurane and sevoflurane anesthesia in brains of the migratory locusts may also affect their recovery from anesthesia. Given the findings that the EC50 of isoflurane and sevoflurane on the migratory locusts were 1.2% and 3.2%, respectively, we applied RNA-seq to assess gene expression profiles in brains of the migratory locusts after being exposed to isoflurane (1.2%) and sevoflurane (3.2%). RNA-seq analysis showed that 20 differentially expressed transcripts (DETs) were upregulated and 125 DETs were downregulated after exposure to 30-min isoflurane (1.2%) (Figures 3D,F). On the other hand, after 30-min sevoflurane exposure, 13 DETs were upregulated and 5 DETs were downregulated significantly in brains of the migratory locusts (Figures 3E,F). Cluster analysis showed the differential expression patterns in responding to isoflurane and sevoflurane anesthesia (Figure 3G). The isoflurane (1.2%) and sevoflurane (3.2%) induce nearly 50% of the migratory locusts in anesthesia, and there was no other obvious differentiation in phenotypes in responding to isoflurane and sevoflurane anesthesia. Although transcripts were expressed differentially in responding to isoflurane and sevoflurane anesthesia, and there may be no causal relationship between differential transcript expression and anesthetic responses. However, the recovery duration is shorter in sevoflurane anesthesia (3.2%) than in isoflurane anesthesia (1.2%). Thus, the genome-wide differential expression patterns of those transcripts induced by isoflurane and sevoflurane anesthesia may affect the differential recovery from isoflurane and sevoflurane anesthesia.

### The Critical Transcripts May Mediate the Differential Recovery Between Isoflurane and Sevoflurane Anesthesia

**FIGURE 4:**
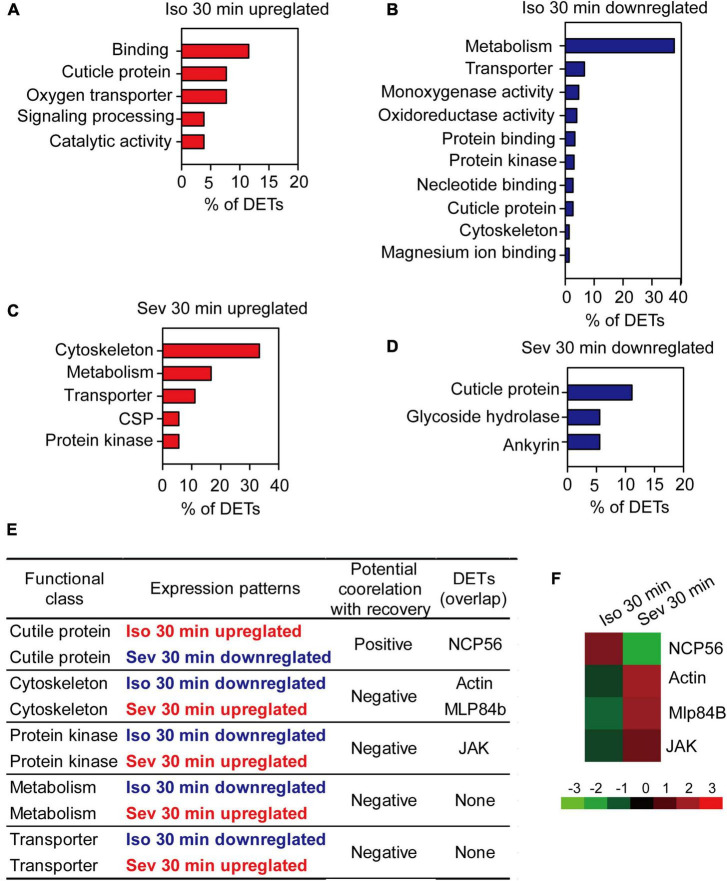
The pivotal genes potentially involved in post-anesthesia recovery in brains of the migratory locusts. **(A,B)** Functional classes of upregulated **(A)** and downregulated **(B)** DETs induced by 30 min of isoflurane exposure. **(C,D)** Functional classes of upregulated **(C)** and downregulated **(D)** DETs induced by 30 min of sevoflurane exposure. **(E)** The functional classes potentially correlated with post-anesthesia recovery from isoflurane and sevoflurane. **(F)** Expression levels of the four pivotal transcripts potentially involved differential recovery from isoflurane and sevoflurane anesthesia. Mlp84B, Myosin-like protein 84B; NCP56, nymph cuticle protein 56; JAK, Janus kinase; Iso, isoflurane; Sev, sevoflurane.

In this study, behavioral phenotype analysis revealed no difference between the percentage of isoflurane-anesthetized and sevoflurane-anesthetized locusts. By contrast, the recovery duration from sevoflurane anesthesia was shorter than that from isoflurane anesthesia. Thus, the differentially expressed transcripts (DETs) resulting from isoflurane or sevoflurane anesthesia may independently or cooperatively mediate the recovery duration from isoflurane and sevoflurane anesthesia. After functional classification analysis of those upregulated or downregulated DETs resulting from isoflurane or sevoflurane anesthesia, we presented the upregulated and downregulated gene classes in Figure 4.

Considering that recovery duration from isoflurane anesthesia was longer than that from sevoflurane anesthesia, the gene classes upregulated in isoflurane anesthesia and downregulated in sevoflurane anesthesia may be positively correlated with longer duration of recovery from isoflurane and shorter duration of recovery from sevoflurane, whereas the gene classes downregulated in isoflurane anesthesia and upregulated in sevoflurane anesthesia may be negatively correlated with longer duration of recovery from isoflurane anesthesia and shorter duration of recovery from sevoflurane anesthesia. Thus, the gene class of cuticle proteins upregulated in isoflurane anesthesia and downregulated in sevoflurane anesthesia may be positively correlated with the differential recovery duration from isoflurane and sevoflurane anesthesia (Figures 4A,D,E), whereas those gene classes cytoskeleton, protein kinase, metabolism and transporter that were downregulated in isoflurane anesthesia and upregulated in sevoflurane anesthesia may be negatively correlated with differential recovery duration from isoflurane and sevoflurane anesthesia (Figures 4B,C,E). Transcripts in these gene classes may be involved in modulating differential recovery from isoflurane or sevoflurane anesthesia.

Among the transcripts in functional classes correlated with recovery from isoflurane or sevoflurane anesthesia, some transcripts were differentially expressed solely after isoflurane and sevoflurane exposure, and these transcripts may independently mediate isoflurane recovery or sevoflurane recovery. Moreover, the other transcripts were differentially expressed both in isoflurane and sevoflurane anesthesia, and these overlapped transcripts may be involved in differential recovery from isoflurane and sevoflurane anesthesia. Thus, from the following five gene classes cuticle protein, cytoskeleton, metabolism, protein kinase and transporter that were positively or negatively correlated with the differential recovery from isoflurane and sevoflurane anesthesia, we found that four transcripts nymphal cuticle protein 56 (*NCP56*), *Actin*, *Mlp84B* and *JAK* were closely correlated with differential recovery from isoflurane and sevoflurane anesthesia (Figures 4E,F). Although we finally focused on these four transcripts for further functional validation in differential recovery from isoflurane and sevoflurane anesthesia, we did not intend to deny the functions of the remaining DETs specifically resulting from isoflurane or sevoflurane anesthesia. These remaining DETs may solely mediate the recovery from isoflurane anesthesia or sevoflurane anesthesia. Thus, *Actin*, *Mlp84B*, *JAK* and *NCP56* may potentially mediate the differential recovery from isoflurane and sevoflurane anesthesia.

**FIGURE 5:**
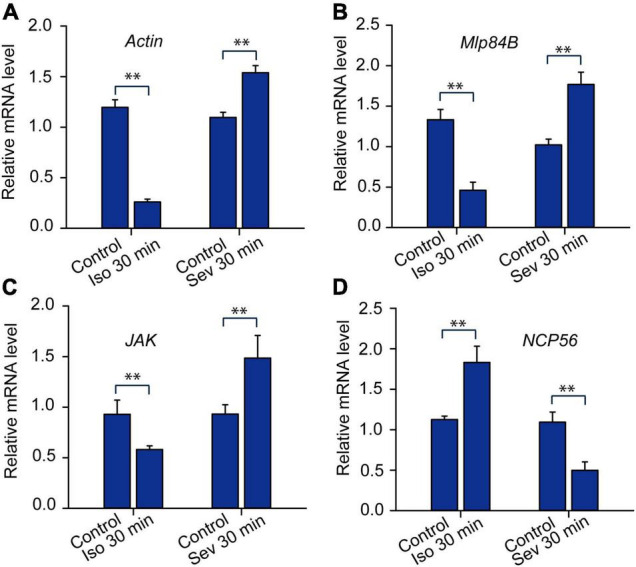
The expression profiles of the transcripts expressed differentially in responding to isoflurane (1.2%) and sevoflurane (3.2%) anesthesia. The expression levels of *Actin* (**A,** *n* = 6), *Mlp84B* (**B,** *n* = 6), *JAK* (**C,** *n* = 6) and *NCP56* (**D**, *n* = 6) in brains of the migratory locusts in responding to 30 min of isoflurane and sevoflurane, respectively. Student’s *t*-test is used for analyzing gene expression levels underlying anesthesia. The expression levels of those genes are shown as the mean ± SEM. ^**^P < 0.01. Mlp84B, Myosin-like protein 84B; NCP56, nymph cuticle protein 56; JAK, Janus kinase; Iso, isoflurane; Sev, sevoflurane.

Moreover, we applied qRT-PCR to analyze expression patterns of *Actin*, *Mlp84B*, *JAK* and *NCP56* after isoflurane and sevoflurane exposure. The results showed that expression patterns of *Actin*, *Mlp84B* and *JAK* were decreased after isoflurane anesthesia (*Actin*: *t* = 5.08, *P* < 0.01; *Mlp84B: t* = 4.454, *P* < 0.01; JAK: *t* = 5.308, *P* < 0.01), but increased after sevoflurane anesthesia (*Actin*: *t* = 11.576, *P* < 0.01; *Mlp84B: t* = 5.421, *P* < 0.01; *JAK*: *t* = 3.334, *P* < 0.01). By contrast, the expression level of *NCP56* was increased after isoflurane anesthesia (*t* = 3.431, *P* < 0.01), but decreased after sevoflurane anesthesia (*t* = 3.650, *P* < 0.01) (Figure 5). These results suggested that expression levels of the four transcripts underlying anesthetics exposure may be associated with differential recovery from isoflurane and sevoflurane anesthesia.

### Janus Kinase Mediates the Shorter Recovery From 30-Min Isoflurane Anesthesia Than 30-Min Sevoflurane Anesthesia

**FIGURE 6:**
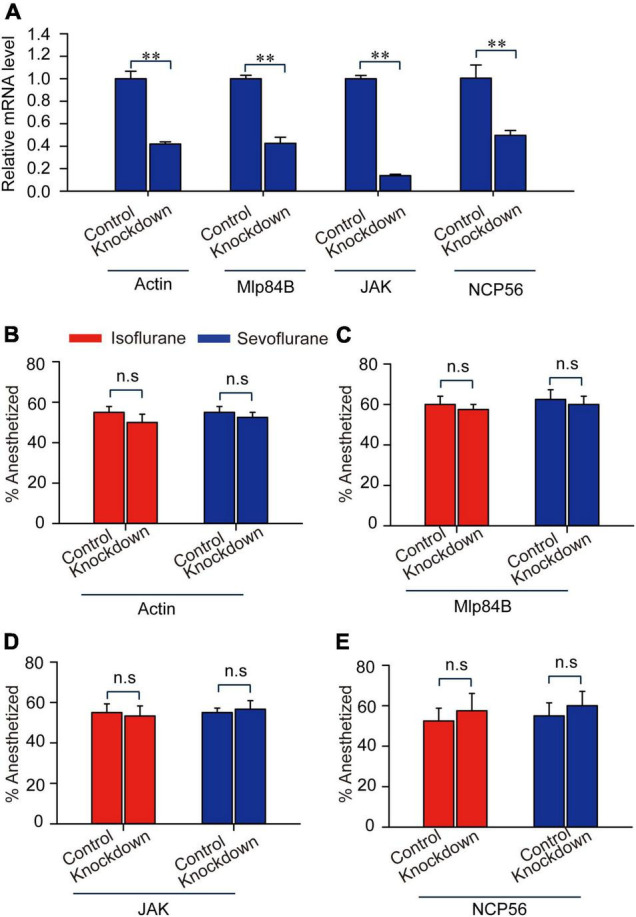
*Actin*, *Mlp84B*, *JAK* and *NCP56* do not affect anesthetic responses of the migratory locusts to isoflurane and sevoflurane. **(A)** The expression levels of *Actin*, *Mlp84B*, *JAK* and *NCP56* mRNA are decreased significantly after RNAi knockdown (*n* = 6 for each gene). **(B–E)** RNAi knockdown of *Actin* **(B)**, *Mlp84B* **(C)**, *JAK* **(D)**, and *NCP56* **(E)** (*n* = 40 for each treatment) do not affect the percentage of anesthetized locusts in response to isoflurane and sevoflurane, respectively. Student’s *t*-test is used for analyzing gene expression levels before and after RNAi knockdown, and anesthesia effects respectively. The percentage of anesthetized locusts is presented as mean ± SEM. n.s *P* > 0.05; ^**^P < 0.01. Mlp84B, Myosin-like protein 84B; NCP56, nymph cuticle protein 56; JAK, Janus kinase.

To validate the functions of those four transcripts *Actin*, *Mlp84B*, *JAK*, and *NCP56* in modulating anesthetic responses to isoflurane and sevoflurane, we designed double-stranded RNA (dsRNAs) of these four transcripts, knocked down expression levels of them and analyzed their expression levels by qRT-PCR. The results showed that expression levels of these four transcripts were significantly decreased after RNAi knockdown (*JAK*: *t* = 13.912, *P* < 0.01; *Mlp84B: t* = 8.3, *P* < 0.01; *Actin*: *t* = 9.195, *P* < 0.01; *NCP56*: *t* = 4.082, *P* < 0.01) (Figure 6A). Considering that expression levels of these four transcripts were expressed differentially after 30-min sevoflurane and isoflurane exposure, respectively, we then confirmed their functions involved in post-anesthesia recovery. The results showed that the percentage of anesthetized locusts did not change after RNAi knockdown of *Actin*, *Mlp84B*, *JAK*, and *NCP56*, respectively, as compared with the control group injected with double-stranded RNA of GFP (dsGFP) (*Actin*: *t* = 1.00, *P* = 0.356; *Mlp84B*: *t* = 0.522, *P* = 0.62; *JAK*: *t* = 0.255, *P* = 0.804; *NCP56*: *t* = 0.471, *P* = 0.564) and sevoflurane anesthesia (*Actin*: *t* = 0.655, *P* = 0.537, *Mlp84B*: *t* = 0.397, *P* = 0.705; *JAK*: *t* = 0.349, *P* = 0.734; *NCP56*: *t* = 0.522, *P* = 0.620) (Figures 6B–E), suggesting that these four transcripts are not involved in modulating anesthetic responses.

**FIGURE 7:**
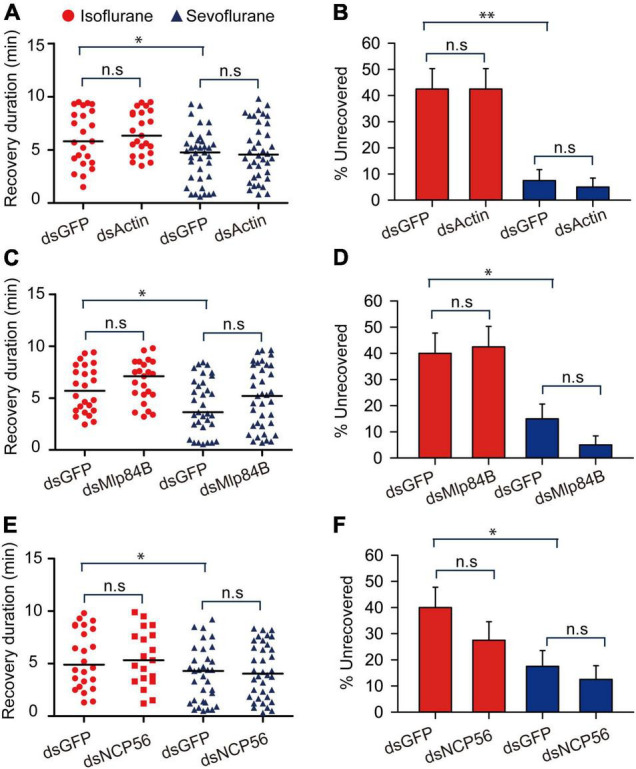
*Actin*, *Mlp84B*, *JAK* and *NCP56* do not affect recovery duration from isoflurane and sevoflurane anesthesia. **(A)** *Actin* RNAi knockdown does not respectively affect the duration of recovery from isoflurane and sevoflurane anesthesia (*n* = 40 for each treatment). **(B)** *Actin* RNAi knockdown does not affect the percentage of unrecovered locusts with isoflurane and sevoflurane anesthesia (*n* = 40 for each treatment). **(C)** The effects of *Mlp84B* RNAi knockdown on the recovery duration from isoflurane and sevoflurane anesthesia (*n* = 40 for each treatment). **(D)** *Mlp84B* RNAi knockdown does not affect the percentage of unrecovered locusts with isoflurane and sevoflurane anesthesia (*n* = 40 for each treatment). **(E)** *NCP56* RNAi knockdown does not affect the recovery duration from isoflurane and sevoflurane anesthesia (*n* = 40 for each treatment). **(F)** *NCP56* RNAi knockdown does not affect the percentage of unrecovered locusts with isoflurane and sevoflurane anesthesia (*n* = 40 for each treatment). Mann–Whitney *U* test is used for analyzing differential recovery duration from isoflurane and sevoflurane anesthesia, and G-test for independence is used for analyzing the effects of RNAi knockdown on the percentage of uncovered locusts within10 min after discontinuing anesthesia. n.s *P* > 0.05, ^**^P < 0.01, *P < 0.05. Abbreviations: Mlp84B, Myosin-like protein 84B; NCP56, nymph cuticle protein 56.

Considering that the recovery duration of 30-min sevoflurane anesthesia is shorter than that of 30-min isoflurane anesthesia, we then explored the functional relationship of these four transcripts with duration of recovery from isoflurane and sevoflurane anesthesia in the migratory locusts. Although we found recovery duration from sevoflurane anesthesia is shorter than isoflurane anesthesia in dsGFP-injected locusts (*Actin*: Mann–Whitney *U* = 279, *P* = 0.026; *Mlp84B:* Mann–Whitney *U* = 258, *P* = 0.0256; *NCP56*: Mann–Whitney *U* = 273.5, *P* = 0.0476), the three transcripts *Actin*, *Mlp84B*, and *NCP56* did not affect the recovery duration from isoflurane anesthesia (*Actin*: Mann–Whitney *U* = 234, *P* = 0.503; *Mlp84B:* Mann–Whitney *U* = 218.5, *P* = 0.221; *NCP56*: Mann–Whitney *U* = 224, *P* = 0.932) and sevoflurane anesthesia (*Actin:* Mann–Whitney *U* = 647, *P* = 0.533; *Mlp84B*: Mann–Whitney *U* = 505, *P* = 0.214; *NCP56:* Mann–Whitney *U* = 557.5, *P* = 0.331) within 10 min after discontinuing anesthesia (Figures 7A,C,E). Moreover, the percentage of unrecovered locusts is higher in isoflurane anesthesia than in sevoflurane anesthesia in dsGFP-injected locusts (*Actin*: *G* = 14.11, *P* < 0.001; *Mlp84B: G* = 6.449, *P* = 0.011; *NCP56*: *G* = 5.04, *P* = 0.024), but knockdown of these three transcripts did not affect the percentage of unrecovered locusts from isoflurane anesthesia (*Actin*: *G* = 0.11, *P* = 0.91; *Mlp84B: G* = 0.05, *P* = 0.82; *NCP5*6: *G* = 0.08, *P* = 0.77) or sevoflurane anesthesia (*Actin*: *G* = 0.21, *P* = 0.64; *Mlp84B: G* = 2.31, *P* = 0.128; *NCP56*: *G* = 0.39, *P* = 0.53) (Figures 7B,D,F).

**FIGURE 8:**
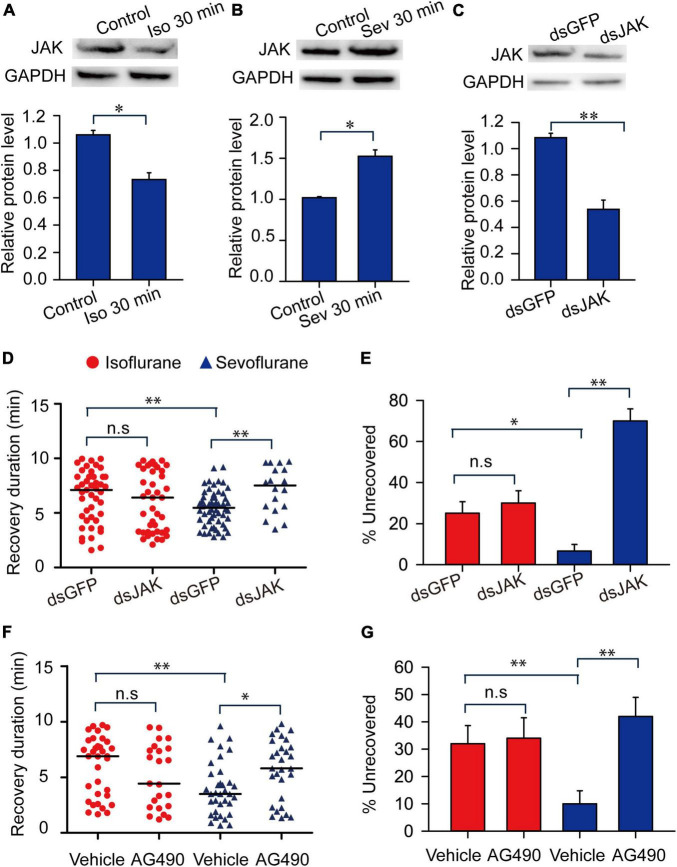
Janus kinase mediates the shorter duration of recovery duration from sevoflurane anesthesia than isoflurane anesthesia. **(A)** Isoflurane anesthesia induces the decrease of the expression level of JAK protein (*n* = 6). **(B)** Sevoflurane anesthesia induces the increase of the expression level of JAK protein (*n* = 6). **(C)** *JAK* RNAi knockdown results in the decrease of JAK protein levels in brains of the migratory locusts (*n* = 6). **(D,E)** *JAK* RNAi knockdown **(D)** results in longer duration of recovery from sevoflurane anesthesia, and increases the percentage of unrecovered locusts with sevoflurane anesthesia **(E)** within 10 min after discontinuing anesthesia (*n* = 40 for each treatment). **(F,G)** Injection of JAK inhibitor AG490 **(F)** results in the longer duration of recovery from sevoflurane anesthesia, and increases the percentage of unrecovered locusts with sevoflurane anesthesia **(G)** within 10 min after discontinuing anesthesia (*n* = 40 for each treatment). Student’s *t*-test is performed to analyze statistical significance of JAK expression levels compared with the corresponding controls. Mann–Whitney *U* test is used for analyzing differential recovery duration from isoflurane and sevoflurane anesthesia, and G-test for independence is used for analyzing the effects of JAK deficiency on the percentage of unrecovered locusts within 10 min after discontinuing anesthesia. n.s *P* > 0.05, ^**^P < 0.01, *P < 0.05. JAK, Janus kinase, ISO, isoflurane, SEV, sevoflurane, STAT5B, signal transducer and activator of transcription 5B.

We further analyzed expression levels of JAK protein in response to isoflurane and sevoflurane exposure and explored the functional correlation of JAK with anesthetic recovery from isoflurane and sevoflurane anesthesia. The results showed that 30-min isoflurane anesthesia induced a decrease of the expression level of JAK protein in brains (*t* = 2.41, *P* < 0.05) (Figure 8A), whereas 30-min sevoflurane anesthesia induced an increase of JAK protein level (*t* = 2.288, *P* < 0.05) (Figure 8B). Moreover, JAK RNAi knockdown in brains resulted in a significant decrease of its protein expression level (*t* = 3.818, *P* < 0.01) (Figure 8C). Considering that JAK RNAi knockdown did not affect the percentage of the migratory locust with isoflurane and sevoflurane anesthesia (*t* = 0.302, *P* > 0.05 for isoflurane; *t* = 0.148, *P* > 0.05 for sevoflurane), we then injected JAK inhibitor AG490 in locusts and further validated that this inhibitor did not affect anesthetic responses to isoflurane or sevoflurane exposure (*t* = 0.361, *P* = 0.730 for isoflurane; *t* = 1.00, *P* = 0.356 for sevoflurane) (Supplementary Figure 1A). Moreover, JAK RNAi knockdown did not affect the recovery from isoflurane anesthesia (Mann–Whitney *U* = 834, *P* = 0.44), whereas deficiency of JAK by RNAi knockdown resulted in a longer duration of recovery from sevoflurane anesthesia within 10 min after discontinuing anesthesia (Mann–Whitney *U* = 270, *P* = 0.003) (Figure 8D). The injection of dsGFP in locusts did not affect longer duration of recovery from isoflurane anesthesia than that recovery from sevoflurane anesthesia (Mann–Whitney *U* = 885, *P* = 0.01) (Figure 8D). On the other hand, within 10 min after discontinuing isoflurane or sevoflurane anesthesia, dsGFP injection did not affect the lower percentage of uncovered locusts with sevoflurane anesthesia than those with isoflurane anesthesia (*G* = 7.98, P = 0.005) (Figure 8E). Although JAK RNAi knockdown did not significantly change the percentage of unrecovered locusts with isoflurane anesthesia (*G* = 0.37, *P* = 0.54), this knockdown significantly increased the percentage of unrecovered locusts with sevoflurane anesthesia (*G* = 57, *P* < 0.001) (Figure 8E).

To further validate the function of JAK in modulating recovery from sevoflurane anesthesia, we injected JAK inhibitor AG490 in head cavity of the migratory locusts and found that JAK inhibitor AG490 did not affect the duration of recovery from isoflurane anesthesia (Mann–Whitney *U* = 308, *P* = 0.177), and this inhibitor induced a longer duration of recovery from sevoflurane anesthesia (Mann–Whitney *U* = 302.5, *P* = 0.013) within 10 min after discontinuing anesthesia (Figure 8F). Moreover, the recovery duration of the vehicle-injected locusts with isoflurane exposure is longer than those vehicle-injected insects with sevoflurane exposure (Mann–Whitney *U* = 337.5, *P* = 0.0051) (Figure 8F). On the other hand, after 10 min of anesthesia, the percentage of unrecovered insects with vehicle injection and isoflurane anesthesia is higher than those of vehicle-injected insects with sevoflurane anesthesia (*G* = 4.96, *P* = 0.026). AG490 injection did not change the percentage of these unrecovered locusts exposed to isoflurane anesthesia, as compared with vehicle controls (*G* = 1.05, *P* = 0.305) (Figure 8G). By contrast, this injection significantly increased the percentage of unrecovered locusts with sevoflurane anesthesia (*G* = 10.037, *P* = 0.002) (Figure 8G). These results indicated that JAK mediates the shorter duration of recovery from sevoflurane anesthesia than isoflurane anesthesia.

**FIGURE 9:**
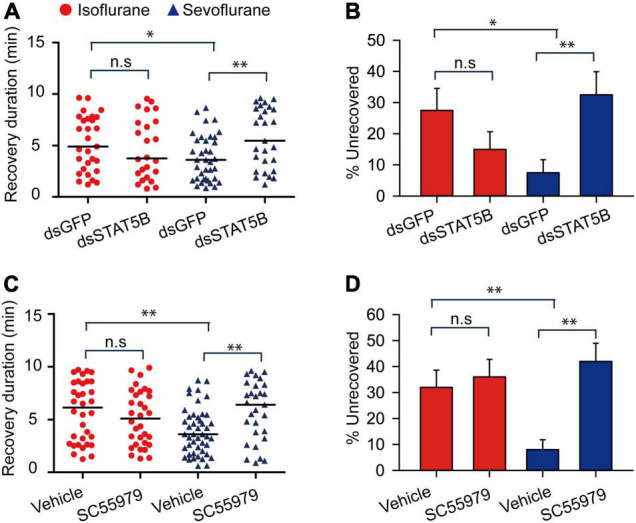
STAT5B mediates the shorter duration of recovery from sevoflurane anesthesia than isoflurane anesthesia. **(A,B)** *STAT5B* RNAi knockdown **(A)** results in longer duration of recovery from sevoflurane anesthesia, and increases the percentage of unrecovered locusts with sevoflurane anesthesia **(B)** within 10 min after discontinuing anesthesia (*n* = 40 for each treatment). **(C,D)** Injection of STAT5B inhibitor SC55979 **(C)** results in longer duration of recovery from sevoflurane anesthesia, and increases the percentage of unrecovered locusts with sevoflurane anesthesia **(D)** within 10 min after discontinuing anesthesia (*n* = 40 for each treatment). Mann–Whitney *U* test is used for analyzing differential recovery duration from isoflurane and sevoflurane anesthesia, and *G-test for independence* is used for analyzing the effects of JAK deficiency on the percentage of unrecovered locusts within 10 min after discontinuing anesthesia. n.s *P* > 0.05, ^**^P < 0.01, *P < 0.05. ISO, isoflurane, SEV, sevoflurane, STAT5B, signal transducer and activator of transcription 5B.

Janus kinase activates signal transducer and activator of transcription 5B (STAT5B) to mediate various cell activities in the migratory locusts (Miljus et al., 2014). Thus, we further explored STAT5B functions in mediating anesthesia recovery and presented more evidences to validate JAK functions in mediating anesthesia recovery. After knocking down expression levels of STAT5B mRNA (*t* = 3.641, *P* < 0.01) (Supplementary Figure 2), we analyzed anesthetic responses of the migratory locusts to isoflurane and sevoflurane anesthesia. The results showed that STAT5B RNAi knockdown and inhibitor SC355979 induced no obvious effects on anesthetic responses to isoflurane and sevoflurane exposure (STAT5B RNAi knockdown: *t* = 0.397, *P* = 0.705 for isoflurane; *t* = 0.192, *P* = 0.278 for sevoflurane; Inhibitor, *t* = 0.311, *P* = 0.766 for isoflurane; *t* = 0.333, *P* = 0.750 for sevoflurane) (Supplementary Figures 1B,C). Within 10 min after discontinuing anesthesia, STAT5B RNAi knockdown did not affect the recovery duration from isoflurane (Mann–Whitney *U* = 695, *P* = 0.312), but elongated the recovery duration from sevoflurane anesthesia (Mann–Whitney *U* = 502, *P* = 0.004) (Figure 9A). The dsGFP-injected locusts still showed longer duration of recovery from isoflurane anesthesia than those from sevoflurane anesthesia (Mann–Whitney *U* = 532.5, *P* = 0.01) (Figure 9A). Moreover, the percentage of unrecovered locusts with dsGFP injection and isoflurane anesthesia is higher than those with sevoflurane anesthesia (*G* = 5.83, *P* = 0.016) (Figure 9B). Although STAT5B RNAi knockdown did not change the percentage of unrecovered locusts with isoflurane anesthesia (*G* = 0.53, *P* = 0.47), the percentage of unrecovered insects with sevoflurane anesthesia was increased significantly after STAT5B RNAi knockdown (*G* = 8.31, *P* = 0.004) (Figure 9B).

On the other hand, to further validate the function of STAT5B in mediating locust anesthesia recovery, we injected STAT5B inhibitor SC355979 in locust head cavity and found that this inhibitor showed similar functional role to STAT5B RNAi knockdown (Mann–Whitney *U* = 475, *P* = 0.376 for isoflurane; Mann–Whitney *U* = 407.5, *P* = 0.005 for sevoflurane) (Figure 9C), and vehicle injection did not affect longer duration of recovery of locusts from isoflurane anesthesia than those from sevoflurane anesthesia (Mann–Whitney *U* = 497.5, *P* = 0.006) (Figure 9C). Moreover, the percentage of unrecovered locusts with sevoflurane anesthesia and vehicle injection was still significantly lower than those with isoflurane anesthesia and vehicle injection (*G* = 9.517, *P* = 0.002) (Figure 9D). After 10 min of anesthesia, SC355979 injection did not change the percentage of unrecovered locusts with isoflurane anesthesia, but this injection significantly increased the percentage of unrecovered locusts with sevoflurane anesthesia as compared with vehicle-injected groups (*G* = 16.561, *P* < 0.001) (Figure 9D). These results suggest that STAT5B mediates shorter duration of recovery from sevoflurane anesthesia.

## Discussion

In this study, we have found, for the first time, that the migratory locusts show anesthetic responses to isoflurane and sevoflurane, and anesthetic recovery is faster from sevoflurane anesthesia than that from isoflurane anesthesia. This study also shows differential expression patterns of transcripts in responses to isoflurane and sevoflurane anesthesia, respectively. Notably, JAK mediates the shorter duration of recovery from sevoflurane but not from isoflurane anesthesia in the migratory locusts. Thus, this study confirms that JAK mediates faster recovery from sevoflurane anesthesia, and the migratory locusts can be used as an animal system to study the mechanism of recovery from anesthesia.

The migratory locust model shows similar anesthesia tendency and recovery to those of other inveterate models, mammalian models and humans. Previously, the invertebrates for anesthesia study are fruit flies and worms (Zalucki and van Swinderen, 2016; Fischer et al., 2018). Here the migratory locusts show immobility after isoflurane and sevoflurane exposure and lose the sensation of mechanical stimuli. Moreover, recovery from sevoflurane anesthesia is faster than that from isoflurane anesthesia. The traits of anesthetic response and post-anesthesia recovery in the migratory locusts are similar to those in other invertebrate models and mammals. The migratory locust is a traditional animal model for studying conserved genetic mechanism underlying insect behavioral and neurobiological phenotypes. To study the genetic mechanism underlying anesthesia and post-anesthesia recovery, fruit flies and worms are indeed paramount choices (Zalucki and van Swinderen, 2016; Fischer et al., 2018). However, this insect is easy to culture in the lab, and its larger body size makes it promise for anesthesia behavioral phenotype observation than fruit flies and worms, and its brain size is relatively large that may be more appropriate for pharmacological and neurophysiological manipulation *in vitro*. More importantly, in this locust model, clustered regularly interspaced short palindromic repeats/CRISPR-associated sequence (CRISPR/Cas) system has been successfully used for gene knockout study (Li et al., 2016). The CRISPR activation system (Long et al., 2015) may also be practical for gene overexpression in the near future in the migratory locusts. Thus, the migratory locust shows a prospective future in studying the neurophysiological and genetic mechanisms underlying anesthesia and post-anesthesia recovery.

Here in this study, the migratory locusts show anesthetic responses to isoflurane and sevoflurane, and the recovery duration from sevoflurane anesthesia is shorter than that for isoflurane anesthesia. In previous studies, recovery duration from sevoflurane is shorter than those durations from isoflurane anesthesia (Golembiewski, 2004; Gupta et al., 2004; Arar et al., 2005; Olufs et al., 2018). Moreover, the recovery duration in veterinary clinics is significantly shorter for sevoflurane anesthesia than that for isoflurane anesthesia (Olthoff and Rohrbach, 1998; Schwender et al., 1998). Although the migratory locusts showed similar anesthetic responses to sevoflurane and isoflurane, the recovery duration for sevoflurane after 30-min exposure was shorter than that for isoflurane anesthesia, consistent with the data obtained from the other studies (Ebert et al., 1998). Specifically, postoperative analysis of the effects of isoflurane and sevoflurane anesthesia has shown the relatively faster recovery time from sevoflurane than that from isoflurane anesthesia (Gupta et al., 2004). In rhesus macaque (*Macaca mulatta*), the recovery duration from sevoflurane anesthesia is shorter than that from isoflurane anesthesia (Bertrand et al., 2017). Therefore, the different durations of recovery from isoflurane and sevoflurane in the migratory locusts are consistent with the results noted in other animal systems and in clinical trials.

This study determines that the migratory locusts display anesthetic responses to the tested general anesthetics in a concentration-dependent manner. At each concentration, not all animals could be put down and show inter-individual differences in response to isoflurane or sevoflurane anesthesia. The individual sensitivity to general anesthetics in patients generally occur in clinical practice, but the underlying genetic mechanism remains largely unknown. Although there is a possibility of gas-blood exchange equilibrium during exposure to anesthetics in locusts, inter-individual difference at the genetic level may also contribute to the variation of sensitivity to volatile anesthetics. The sensitivity to general anesthetics may be different in patients (Erden et al., 2018), and these difference may result from the differences in metabolite profile and genetic variations in humans (Wei et al., 2021). Exploration of genetic mechanism underlying inter-individual difference to general anesthetics in the migratory locusts will be beneficial for understanding individual variation of anesthetic sensitivity in clinical practice. Moreover, sevoflurane does not entirely anesthetize the migratory locusts (Figures 1, 2), the stronger sevoflurane anesthesia than we perform in this study may induce more upregulated and downregulated DETs to indicate stronger anesthesia effects as well as stronger toxicity. Exploration of the stronger effects of stronger anesthesia on gene expression regulation and phenotypes will be also helpful in understanding the molecular mechanism of anesthesia recovery.

Although isoflurane (1.2%) and sevoflurane (3.2%) anesthesia induce the similar percentage of anesthetized locusts, the DET numbers and expression patterns are different between isoflurane and sevoflurane exposures, suggesting differential anesthesia mechanism of locusts responding to isoflurane and sevoflurane anesthesia. Isoflurane and sevoflurane interact with GABA and GluR receptors to mediate neurotransmission underlying anesthesia. The post-anesthesia state is therefore the basis for differential recovery from isoflurane and sevoflurane anesthesia. Previous studies reported that isoflurane and sevoflurane take the similar effects on GABA receptors in anesthesia, whereas sevoflurane may play adverse effects with isoflurane on glutamate release in the terminal of pre-synapse (Taylor et al., 2013; Westphalen et al., 2013; Aksenov et al., 2019). These differential effects on neurotransmission system may result in differential speed of recovery from isoflurane and sevoflurane anesthesia.

In addition, sevoflurane exposure induces the upregulation of JAK mRNA and protein levels, whereas isoflurane exposure downregulates expression level of JAK mRNA. JAK does not affect isoflurane and sevoflurane anesthesia, but affects anesthesia recovery from sevoflurane and isoflurane anesthesia. The expression patterns of JAK mRNA and protein in response to isoflurane and sevoflurane anesthetics are potentially related to the mechanism underlying differential recovery from isoflurane and sevoflurane anesthesia in the migratory locusts. Previous studies have reported that sevoflurane shows high affinity for nicotinic acetylcholine receptor (nAchR) and interferes with cholinergic nicotinic neurotransmission (Violet et al., 1997; Tassonyi et al., 2002). JAK acts as a nicotinic acetylcholine receptor (nAchR) target in activating neurostimuli. The nAchR may activate JAK signaling to promote recovery from sevoflurane anesthesia (Shaw et al., 2002; Wiklund et al., 2008). The activation of JAK by this receptor mediates neuroprotection against amyloid-β-(1-42) in Alzheimer’s disease (Shaw et al., 2002; Wiklund et al., 2008). Moreover, JAK signaling pathway protects against behavioral impairments and behavioral learning deficiency (Si et al., 2016) and sevoflurane may activate JAK pathway to protect against hepatic ischemia/reperfusion injury in rats (Sima and Ma, 2019). Notably, sevoflurane activates JAK pathway to exert anti-apoptosis and protect brains from neurotoxicity (Kim et al., 2017). The molecular mechanism of sevoflurane-induced neuroprotection is complex and possibly associated with activation of the canonical Notch signaling pathway, protein kinase C, protein kinase M, tyrosine kinase, extracellular signal-regulated protein kinase (MEK-ERK1/2, ERK1/2 MAPK), and mitochondrial adenosine troposphere-regulated potassium (KATP) channel (Shan et al., 2015). Therefore, JAK may mediate duration of recovery from sevoflurane anesthesia through neuroprotection mechanism that needs to be investigated in the future.

In this study, sevoflurane anesthesia upregulates the expression level of JAK, whereas isoflurane anesthesia downregulates its expression level. However, there is little known about the mechanism that anesthesia exposure mediates expression level of JAK. Sevoflurane mediates the activation of protein kinase A (PKA) pathway in cardioprotection, and promotes transcription through activation of ERK1/2 and activator protein-1 (AP-1) in Kupffer cells (Schwer et al., 2010; Li et al., 2021). Thus, there may be some association between sevoflurane-induced gene transcription and PKA, AP-1 and ERK1/2 activation. On the other hand, isoflurane mediates gene expression through miRNAs in brains (Liu et al., 2021), and isoflurane anesthesia induces the disruption of MEK to ERK activation in mouse brain cortex (Whittington et al., 2013; Salort et al., 2019). Thus, sevoflurane and isoflurane may mediate expression level of JAK at transcriptional and post-transcriptional levels. The regulatory mechanism of sevoflurane and isoflurane in activating and repressing gene expression need to be further explored.

## Conclusion

In conclusion, the migratory locust can be used as an animal model to study the mechanisms underlying anesthesia and post-anesthesia recovery. With accumulation of genomic and transcriptomic data (Wei et al., 2009; Wang et al., 2014; Yang et al., 2019; Li et al., 2020), we could therefore study gene expression regulation underlying anesthesia gene expression, anesthetic potency and recovery time induced by isoflurane and sevoflurane exposures. The JAK signaling induced by sevoflurane anesthesia may protect the locust brain from being damaged by anesthetic neurotoxicity, and this protection may shorten the duration of recovery from sevoflurane anesthesia. By contrast, isoflurane anesthesia depresses JAK signaling and alleviates the protective function of JAK signaling from neurotoxic isoflurane exposure. This alleviation may elongate anesthesia state and hinder recovery from isoflurane exposure. The finding that JAK modulates the duration of recovery from anesthesia provides a solid foundation for studying the mechanisms of postoperative recovery associated with sevoflurane and isoflurane in brains of the migratory locusts.

## Data Availability Statement

The datasets presented in this study can be found in online repositories. The names of the repository/repositories and accession number(s) can be found below: The raw reads of all samples analyzed by RNA-seq are available from the NCBI SRA server (accession number: PRJNA717646).

## Author Contributions

LK and ZX conceived the experiments, analyzed the data, and wrote the manuscript. ZM performed the experiments, analyzed the data, and wrote the manuscript. JZ helped to conceive the experiments and wrote the manuscript. TL contributed to the new reagents, analytic tools, and clinical suggestions. All authors contributed to the article and approved the submitted version.

## Conflict of Interest

ZX provided consulting services to Shanghai 9th and 10th Hospitals, and to Baxter International, Inc. (as an invited speaker) in the past 36 months. The remaining authors declare that the research was conducted in the absence of any commercial or financial relationships that could be construed as a potential conflict of interest.

## Publisher’s Note

All claims expressed in this article are solely those of the authors and do not necessarily represent those of their affiliated organizations, or those of the publisher, the editors and the reviewers. Any product that may be evaluated in this article, or claim that may be made by its manufacturer, is not guaranteed or endorsed by the publisher.

## Abbreviations

DETs: differentially expressed transcripts
dsRNAs: double-stranded RNAs
dsGFP: double-stranded RNAs of GFP
dsJAK: double-stranded RNAs of JAK
dsSTAT5B: double-stranded RNAs of STAT5B
dsMlp48B: double-stranded RNAs of Mlp48B
dsActin: double-stranded RNAs of Actin
dsNCP56: double-stranded RNAs of NCP56
EC50: half-maximal effective concentrations
GFP: green fluorescent protein
Iso: isoflurane
JAK: Janus kinase
KATP: adenosine troposphere-regulated potassium
MAC: minimum alveolar concentration
Mlp84B: Myosin-like protein 84B
MMLV: Moloney murine leukemia virus
nAchR: nicotinic acetylcholine receptor
NCP56: nymph cuticle protein 56
PBST: phosphate-buffered saline containing 0.1% Tween-20
qRT-PCR: quantitative real-time PCR
RNAi: RNA interference
RNA-seq: RNA sequencing
RSEM: Robust structural equation modeling
Sev: Sevoflurane
STAT5B: Signal transducer and activator of transcription 5B.
