# Supplementary material for: Janus Kinase Mediates Faster Recovery From Sevoflurane Anesthesia Than Isoflurane Anesthesia in the Migratory Locusts
Source: Front Physiol. 2022 Mar 30;13:806746. doi: 10.3389/fphys.2022.806746 (PMC9006988; doi:10.3389/fphys.2022.806746)
Supplement: Supplementary file 1 [file Data_Sheet_1.docx]

**Supplementary materials for**

**JAK mediates faster recovery from sevoflurane anesthesia than isoflurane anesthesia in the migratory locusts**

Zongyuan Ma^1^, Jialin C Zheng^2^, Tianzuo Li^3^, Zhongcong Xie^4*^, Le Kang^1*^

1. Beijing Institutes of Life Science, Chinese Academy of Sciences, Beijing100101, China
2. Center for Translational Neurodegeneration and Regenerative Therapy, Shanghai Tenth People's Hospital affiliated to Tongji University School of Medicine, Shanghai 200072, China;
3. Department of Anesthesiology, Beijing Shijitan Hospital, Capital Medical University, Beijing100038, China
4. Department of Anesthesia, Critical Care and Pain Medicine, Massachusetts General Hospital and Harvard Medical School, Boston, MA, USA.

*Corresponding authors:

Zhongcong Xie, M.D., Professor

Massachusetts General Hospital

Harvard Medical School

149 13th Street, Room 4310, Charlestown

Massachusetts 02129-2060，USA

E-mail: ZXIE@mgh.harvard.edu

Le Kang, Ph.D., Professor

Beijing Institutes of Life Science, Chinese Academy of Sciences

Datun Road, Chaoyang District

Beijing 100101, China

+86-10-64807219

E-mail: lkang@ioz.ac.cn

**Supplementary Video 1**

This video shows sevoflurane (3.2%) anesthesia induction and recovery in one locust. This video is divided into three sections. The first section shows locust normal behaviors without anesthesia. The second section includes the induction of anesthesia in the locust, which is defined by the loss of locomotion activity, and the absence of any responses in legs, abdomen and antennae after mechanical touching by a brush pen. The third section shows anesthesia recovery in the locust. The plastic box for anesthesia is usually sealed and it is not convenient for anesthesia recovery in the sealed box, we therefore transferred this insect to a new box, as showing in this video. The time point for anesthesia recovery in the locust is when the locust stands on its legs after gross body movements (movements in legs, abdomen and antennae).

**Supplementary figure 1**


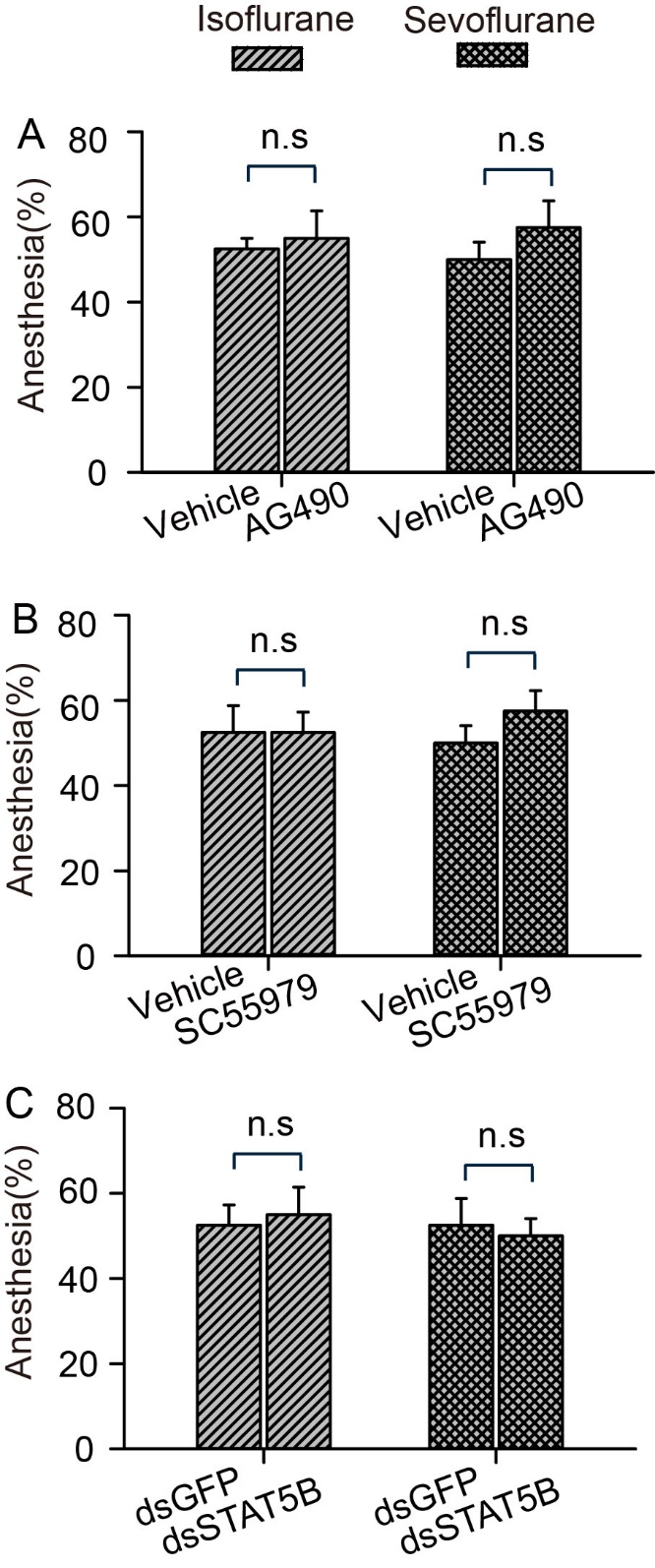


**Supplementary figure 1.** The inhibition of JAK-STAT5B signals does not affect the percentage of locusts in response to isoflurane and sevoflurane anesthesia. (**A**) JAK inhibitor AG490 does not affect the anesthetic responses to isoflurane and the sevolfurane (n =40 for each treatment). (**B and C**) STAT5B inhibitor SC55365 (**B**, n = 40 for each treatment) and STAT5B RNAi knockdown do not affect the anesthetic responses to isoflurane and sevoflurane. Student’s *t*-test is used for analyzing anesthesia effects as compared with corresponding controls. The data are presented as mean ± SEM. n.s *P* > 0.05; ** *P* < 0.01. Abbreviation: STAT5B, signal transducer and activator of transcription 5B.

**Supplementary figure 2**


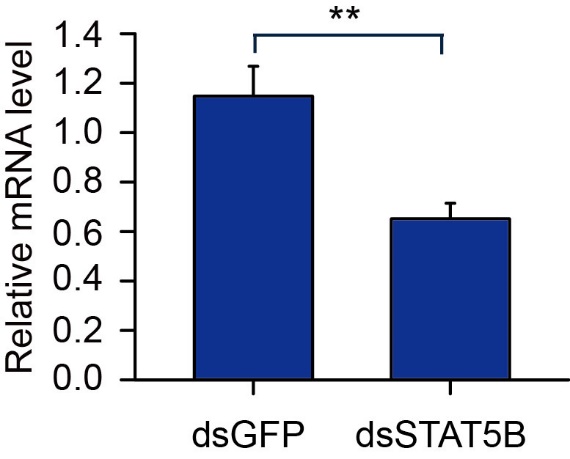


**Supplementary figure 2.** The expression level of STAT5B is significantly decreased after STAT5B RNAi knockdown. Student’s *t*-test is used for analyzing expression level of STAT5B mRNA as compared with corresponding controls. The data are presented as mean ± SEM. ** *P* < 0.01. Abbreviation: STAT5B, signal transducer and activator of transcription 5B.
